# Work Engagement in Serbia: Psychometric Properties of the Serbian Version of the Utrecht Work Engagement Scale (UWES)

**DOI:** 10.3389/fpsyg.2017.01799

**Published:** 2017-10-16

**Authors:** Ivana B. Petrović, Milica Vukelić, Svetlana Čizmić

**Affiliations:** Department of Psychology, Faculty of Philosophy, University of Belgrade, Belgrade, Serbia

**Keywords:** work engagement, The Utrecht Work Engagement Scale (UWES), Serbia, burnout, validity

## Abstract

Work engagement is defined as a positive, affective-motivational state of work-related well-being characterized by vigor, dedication and absorption. The Utrecht Work Engagement Scale (UWES) is the most frequently used measure of work engagement. The aim of this study was to analyze the psychometric properties of the Serbian versions of the UWES-17 and UWES-9. The sample consisted of 860 employees from a number of organizations and jobs across Serbia. Based on the UWES-17 findings, the data confirm both the three-factor and one-factor solutions by giving a slight advantage to the three-factor solution. As for the UWES-9, based on the PCFA and CFA, the one-factor solution was obtained as the preferred one. Taking into account the UWES-9 reliability and correlation patterns of its subscales with other well-being variables, both one- and three-factor solutions of the UWES-9 are suggested for future research. Serbian versions of both the UWES-17 and UWES-9 have satisfactory psychometric properties with high reliability, factorial structure in line with the theoretical model, and good predictive validity. The study contributes to enhanced understanding of work engagement by offering an insight from the Serbian cultural and economic context, significantly different from the UWES originating setting. There is still a need for exploring how employees from Serbia conceptualize work engagement, as well as for further, more stringent investigating of the cultural invariance of the UWES factorial structure.

## Introduction

Since its introduction in 1990 (Bakker, 2017), work engagement has been an inspiration for researchers and practitioners around the world (e.g., Shimazu et al., 2008; Seppälä et al., 2009; Balducci et al., 2010; Nerstad et al., 2010; Fong and Ng, 2012; Littman-Ovadia and Balducci, 2013; Panthee et al., 2014; Zecca et al., 2015; Lovakov et al., 2017). Moreover, it has been regarded as a “societal challenge” (Schaufeli and De Witte, 2017, p. 58) with far reaching effects on the economy. It is widely accepted that, in order to be competitive, contemporary organizations need engaged employees (Bakker, 2017).

As suggested by Schaufeli (2017), based on analyzing the 6^th^ European Working Conditions Survey data from 35 countries, work engagement should be studied not only at the individual level, but also at the national level. Comparing countries with different levels of engagement, Schaufeli demonstrated that work engagement was positively related with national economic activity and productivity. On the European engagement map, the Netherlands, the originating country of the mostly used and cited theoretical model and instrument for assessing work-engagement, the Utrecht Work Engagement Scale (UWES, Schaufeli and Bakker, 2003), was the most engaged country. On the other side, Serbia, the country where the current research takes place, was the least engaged among the 35 surveyed European countries (Eurofound, 2016).

Since the 1990s, Serbia has been encountering a deep, long-lasting socioeconomic crisis, strongly influenced by the wars in the former Yugoslavia, UN sanctions and political instability. On the economic scene, the country went through several models of privatization, including a high failure rate of approximately 25% of unsuccessful privatizations (Vujačić and Petrović-Vujačić, 2011). Similar to other world countries, Serbia was exposed to the deep global financial crisis that had a negative impact on employees and their health and well-being across the world (Giorgi et al., 2015; Mucci et al., 2016; Lopez-Valcarcel and Barber, 2017). Continuously struggling with the hostile conditions, workplace in Serbia has been fluctuating with the numbers of new organizations, new business owners, either as a part of privatization or green-field investment, different work contracts, new technologies and work processes. Nevertheless, contemporary Serbian economy is still characterized by low economic activity (Petrović et al., 2017). GDP per capita is among the bottom 6% of the countries covered by the 2015 European Working Conditions Survey (Eurofound, 2016). The unemployment rate is 18.2% among population aged 15–64 (Statistical Office of the Republic of Serbia, 2016). Bearing all this in mind, in this paper, we deal with work engagement concept in the specific Serbian socio-economic context.

Work engagement is defined as a positive, fulfilling, affective-motivational state of work-related well-being (Bakker et al., 2008). It is characterized by vigor – higher levels of energy, mental resilience and investment of effort; dedication – involvement in work and the sense of meaningfulness and enthusiasm, and absorption – full concentration and engrossment in work (Schaufeli et al., 2002, 2006; Bakker, 2017). In a nutshell, work engagement is about giving “hands, head, and heart” at work (Ashforth and Humphrey, 1995, p. 110).

The concept of work engagement is deeply embedded in job demands–resources theory (JD–R), which sees engagement as a function of an interplay between job demands and resources (Bakker and Demerouti, 2007; Bakker, 2017). Work engagement is, thus, a mediator between job demands/resources and job performance. Job resources such as career opportunities, organizational support, job security, and positive work climate lead to high work engagement and, consequently, to outstanding performance. On the other hand, job demands such as high work pressure, poor physical conditions, negative work climate and challenging interpersonal relations are associated with negative psychological consequences. If a person does not pull together the personal and organizational resources in order to recover from pressures at work, job demands can lead to burnout and exhaustion.

The Utrecht Work Engagement Scale (UWES) developed by Arnold Bakker and Wilmar Schaufeli is the most frequently used measure of work engagement (Bakker and Demerouti, 2016). Systematic literature review shows the UWES was almost exclusively applied as a valid basis for developing work engagement interventions (Knight et al., 2017). The UWES is a self-report scale, with 17-item and 9-item versions widely used in independent national research studies all over the world. There is also an emerging three-item version that was applied in the 6^th^ European Working Conditions Survey (Schaufeli, 2017). Both long and short versions of the UWES cover all three theoretically postulated dimensions: vigor, dedication and absorption. In the 9-item version of the UWES each dimension is covered by three items, while in the 17-item version vigor is covered with six items, dedication with five items and absorption with six items (Schaufeli et al., 2002). Since two items of the original 17-item version were considered as problematic (item 6 from the vigor subscale, and item 6 from the absorption subscale), the 15-item version of the UWES has also been applied in research (Schaufeli and Bakker, 2003). The newest three-item version of the UWES covers all three dimensions of work engagement with one item each (Schaufeli, 2017): for vigor ‘At my work I feel full of energy’ (VI1, modified); for dedication ‘I am enthusiastic about my job’ (DE2), and for absorption ‘Time flies when I am working’ (AB1). There are 30 language versions of the UWES, available at the Schaufeli’s web site.

Previous national validation studies gave somewhat conflicting results about the structure of the UWES and its demographic correlates, while findings about well-being correlates have been quite consistent. Besides the initial Dutch version (Schaufeli and Bakker, 2003), confirmatory factor analysis supported theoretical conceptualization of the three-factor structure (vigor, dedication and absorption) for both the UWES-17 and the UWES-9 Finnish (Seppälä et al., 2009) and Norwegian (Nerstad et al., 2010) language versions. The UWES-9 three-factor structure was confirmed in Chinese (Fong and Ng, 2012); French (Zecca et al., 2015); Hebrew (Littman-Ovadia and Balducci, 2013); Italian (Balducci et al., 2010); Nepali (Panthee et al., 2014); Russian (Lovakov et al., 2017), and Swedish (Hallberg and Schaufeli, 2006) language versions. The one factor UWES-17 was found in the Japanese validation study (Shimazu et al., 2008). Moreover, it is interesting to note that based on the data from a large single company sample from South Africa, applying six UWES-17 language versions, i.e., Afrikaans, English, Nguni, Tshivenda (Venda), Sotho and Xitsonga (Tsonga), based on item response modeling, the one-factor solution has also been suggested (Goliath-Yarde and Roodt, 2011; de Bruin et al., 2013). The one factor UWES-9 solution was found in the Brazilian (Ferreira et al., 2016); Chinese (Fong and Ng, 2012); Japanese (Shimazu et al., 2008); Russian (Lovakov et al., 2017), and Swedish (Hallberg and Schaufeli, 2006) UWES versions. By analyzing the data from 10 different countries, Schaufeli et al. (2006) concluded that work engagement was weakly positively related with age, with correlations about 0.15 or less. Furthermore, the differences between men and women in relation to work engagement were ambiguous, with the lack of differences in some countries (Australia, Canada, and France), and low significant differences with low effect size in other countries (e.g., women from Netherlands, Spain, and South Africa showed higher engagement, whereas men from Belgium, Germany, Finland, and Norway expressed higher engagement than women). Managers scored higher on engagement than general staff (Andreassen et al., 2012; Littman-Ovadia and Balducci, 2013; Panthee et al., 2014).

Of all well-being measures, work engagement is usually linked with burnout. There are two different approaches regarding this relationship (Schaufeli and Bakker, 2003). The first one postulates that burnout is the opposite pole of work engagement (Maslach and Leiter, 1997). The second proposes that these constructs should be regarded as different (Schaufeli and Bakker, 2003). Schaufeli and Bakker (2003) support their argument by the thesis that the correlation between these concepts is not full, and that evaluating both concepts with the same instrument is not empirically justified. Some later tendencies postulate the thesis that burnout and engagement constitute ‘dual unity,’ which is at the same time ‘real and redundant’ (Schaufeli and De Witte, 2017, p. 58). Apart from burnout, work engagement factor structure is usually studied in relation with job satisfaction (e.g., Shimazu et al., 2008; Panthee et al., 2014; Lovakov et al., 2017).

The aim the current study was to analyze the psychometric properties of the UWES-S, the Serbian adaptation of the Utrecht Work Engagement Scale (UWES, Schaufeli and Bakker, 2003). In particular, the aims were to: (1) Investigate factorial structure, both for the 17-item and 9-item versions of the UWES-S; (2) Verify theoretically based structure of the long and short versions of the UWES-S, i.e., to compare the theoretically based three-dimension model of vigor, dedication, and absorption with the one-dimension solution supported by some validation studies (e.g., Shimazu et al., 2008); (3) Investigate Cronbach’s Alpha reliability of the different versions of the UWES and corresponding subscales; (4) Investigate the construct validity through the relationship between work engagement and selected well-being indicators (burnout, organizational support, job satisfaction, job insecurity), and (5) Explore work engagement across demographic variables. By doing so, we wanted to deepen our understanding of work engagement by offering an insight from the Serbian cultural and economic setting, specifically different from the Western-European UWES originating setting, and the settings where mainstream work engagement research and validation studies took place.

## Materials and Methods

### Translation

In order to develop the Serbian version of the UWES, we conducted a number of small-scale studies between 2014 and 2017. The first step was translating and adapting the UWES. After consolidating the Serbian adaptation of the UWES, we started collecting data for the present study.

The UWES-17 was translated to Serbian through the committee technique in three iterations (Brislin et al., 1973). In the process of translation, we used English, French, and Russian versions of the UWES (Schaufeli and Bakker, 2003). In each step, we carried out back translation into English. Since some problems in understanding of the items were presumed during the translation phase, the final version was fine-tuned based on the individual interviews and a focus group discussion^1^ with employees with medium and higher education. We asked the employees about their understanding of each item, the subordinate concept, and the concrete examples of situations at work related to each item. Two absorption items proved to be particularly challenging for the production workers: “When I am working, I forget everything else around me” (AB2), and “I get carried away when I’m working” (AB5). They considered absorption as expressed in these items to be dangerous and unsafe work behavior. Hence, the aim was to make these items comprehensible for each professional group. Thus, the back translation of AB2 was “When I am working, I forget everything that is not related to work,” and for AB5 “I get so much into the job when I’m working.” Before the final version was produced, some research studies with the preliminary versions of the UWES were performed (e.g., Mladenović and Petrović, 2015), showing, for example, high reliability, with the Cronbach’s alpha 0.91 for the short version of the UWES-9.

### Participants

The sample consisted of 860 employees (63% women) from a variety of organizations and jobs across Serbia. Participants’ age ranged from 21 to 73 years (*M* = 40; *SD* = 11.44). Majority of respondents had a university diploma (56.7%), 33.0% completed secondary school, 9.7% completed trade school/college, and 0.6% had elementary education. There were 85.9% subordinates and 14.1% participants on managerial or supervisory positions. There was an almost equal number of participants from the state-owned (48.7%) and private organizations (45.3%). Only 5.1% of participants came from an organization with mixed ownership, and 0.8% from civil sector organizations. More than two thirds of the respondents came from the educational sector (27.5%), health sector (19.3%), processing industry (13.2%), and IT sector (8.5%), while the remaining 31.5% came from other sectors. All respondents were formally and permanently employed. Participation was voluntary and not compensated. This study was carried out in accordance with the Code of Ethics of the Serbian Psychological Society, and approved by the Committee on Ethical Issues of the Society of Psychologists of Serbia Ethics Commission at the Department of Psychology, Faculty of Philosophy, University of Belgrade, with written informed consent from all participants.

### Instruments

Work Engagement was assessed by the Serbian version of the Utrecht Work Engagement Scale, UWES-S. The long version of the UWES (Schaufeli and Bakker, 2003) consists of 17 items followed by a seven-point scale (from 0 = *Never*, to 6 = *Always*). The items are divided into subscales: Vigor, Dedication, and Absorption. **Table 1** contains the English wording of the items from Schaufeli and Bakker’s (2003) Manual. The short version of the UWES consists of 9 items, with three items for each of the three factors (**Table 2**, English version of items, Schaufeli and Bakker, 2003).

**Table 1:** Summary of PCFA with promax rotation for the UWES-17 scale.

Items	Rotated Factor Loadings				
	
	*M*	*SD*	Component 1	Component 2	Component 3
(2) I find the work that I do full of meaning and purpose *(DE1)*	4.68	1.38	**0.876**	-0.093	-0.135
(10) I am proud on the work that I do *(DE4)*	4.42	1.56	**0.800**	0.071	-0.026
(13) To me, my job is challenging *(DE5)*	4.15	1.55	**0.739**	0.039	0.028
(7) My job inspires me *(DE3)*	3.90	1.54	**0.651**	0.344	-0.055
(14) I get carried away when I’m working *(AB5)*	4.83	1.11	**0.637**	-0.235	0.458
(11) I am immersed in my work *(AB4)*	4.47	1.28	**0.615**	-0.039	0.346
(5) I am enthusiastic about my job *(DE2)*	3.79	0.96	**0.542**	0.489	-0.128
(6) When I am working, I forget everything else around me *(AB2)*	3.57	1.00	**0.384**	0.225	0.199
(9) I feel happy when I am working intensely *(AB3)*	3.52	1.09	-0.239	**0.809**	0.121
(1) At my work, I feel bursting with energy *(VI1)*	3.86	1.24	0.193	**0.726**	-0.085
(4) At my job, I feel strong and vigorous *(VI2)*	3.76	1.34	0.229	**0.709**	-0.018
(8) When I get up in the morning, I feel like going to work *(VI3)*	3.36	1.63	0.234	**0.676**	-0.072
(16) It is difficult to detach myself from my job *(AB6)*	2.63	1.50	-0.109	**0.649**	0.242
(3) Time flies when I’m working *(AB1)*	4.56	1.29	0.288	**0.390**	0.082
(17) At my work I always persevere, even when things do not go well *(VI6)*	4.59	1.19	0.187	-0.161	**0.728**
(12) I can continue working for very long periods at a time *(VI4)*	4.06	1.39	-0.302	0.351	**0.718**
(15) At my job, I am very resilient, mentally *(VI5)*	3.83	1.33	-0.001	0.15	**0.636**
Eigenvalues			6.73	6.26	3.86


*The highest loading for each item is given in bold face.*

**Table 2:** Summary of PCFA for the UWES-9 scale.

Items	Item-component correlation	Variance explained
(7) My job inspires me *(DE3)*	0.871	0.758
(5) I am enthusiastic about my job *(DE2)*	0.869	0.755
(4) At my job, I feel strong and vigorous *(VI2)*	0.837	0.700
(8) When I get up in the morning, I feel like going to work *(VI3)*	0.784	0.615
(1) At my work, I feel bursting with energy *(VI1)*	0.782	0.611
(10) I am proud on the work that I do *(DE4)*	0.776	0.602
(11) I am immersed in my work *(AB4)*	0.720	0.518
(14) I get carried away when I’m working *(AB5)*	0.636	0.404
(9) I feel happy when I am working intensely *(AB3)*	0.590	0.348


### Well-being Measures

The following measures of well-being were included in this research: burnout, job satisfaction, job insecurity and perceived organizational support. Burnout was assessed by the Serbian adaptation of the Oldenburg Burnout Inventory (OLBI, Demerouti and Bakker, 2008). The first version of the instrument was constructed for measuring burnout among German employees. The English version of the instrument was validated on 2,599 employees from the United States, where it showed acceptable reliability and validity (Halbesleben and Demerouti, 2005). With 16 positively and negatively worded items, the OLBI covers two dimensions of burnout: exhaustion and disengagement from work. Items were assessed on a seven-point rating scale, from 1 (strongly disagree) to 7 (strongly agree). The OLBI was translated to Serbian applying committee technique and back translation (Brislin et al., 1973). The reliability of the OLBI in this research measured with Cronbach’s alpha was 0.81.

The perceived organizational support was assessed using the Serbian translation of the 8-item version of the Survey of Perceived Organizational Support, SPOS, by Eisenberger et al. (1986). The SPOS has been previously used in research on a large sample of employees from Serbia (Vukelić et al., 2015). The SPOS measures employees’ perception of the extent to which an organization cares about their well-being and respects their contribution to its development. Each item is followed by a 7-point Likert scale. The reliability of the SPOS in this research was very high, with Cronbach’s alpha coefficient of 0.97.

The overall job satisfaction and overall job insecurity were assessed by single-item measures. Job satisfaction was assessed through a question “All in all, how satisfied are you with your job?”, followed by a five-point Likert-type scale, ranging from 1 (not satisfied at all), to 5 (completely satisfied). The item has been previously used in job satisfaction research in Serbia (Kovačević and Petrović, 2007). Job insecurity was assessed through a question “All in all, how secure is your job?”, rated on a three-point scale, ranging from 1, meaning that job was insecure, 2, meaning that job was as secure as other jobs, to 3, implying that job was secure. It is ubiquitous that single-item measures are of an inferior validity to scale measures. Nevertheless, it has been shown that one-item job satisfaction measures are of satisfactory validity for assessing general job satisfaction (Wanous et al., 1997). Moreover, they are considered to be more acceptable and face valid in organizational context, less time consuming and thus more cost-effective (e.g., Wanous et al., 1997; Nagy, 2002). The single-item measure of job satisfaction in relation to the UWES scale was previously used in the research of Shimazu and Schaufeli (2009). The one-item measure of job (in)security has been previously used within the JD–R framework by Demerouti et al. (2001) and within the framework of the JD–C (Job Demand–Control Model) by de Jonge et al. (2000).

### Statistical Analysis

Exploratory factor analysis and hierarchical regression analysis were conducted using IBM SPSS 21.0. Confirmatory factor analysis (CFA) of both the 17-item and 9-item versions of the UWES-Serbian was performed in IBM SPSS AMOS 21 using structural equation modeling (SEM). We used the Asymptotically Distribution-free Estimates method to examine goodness of fit of the models as it was more sensitive to non-normal distribution of scores (Benson and Fleishman, 1994; Maydeu-Olivares et al., 2007). The CFA was carried out without cross-loadings or correlation between errors.

## Results

### Factorial Structure of the UWES and Inter-correlations among Factors

In order to reveal the factorial structure of the UWES-17, all items were subjected to principal components factor analysis (PCFA) with promax rotation (**Table 1**, items’ means and standard deviations are also presented). Previously, data were analyzed in order to estimate adequacy for the PCFA. The value of Kaiser-Meyer-Olkin (KMO) measure was 0.940 and all KMO values for the individual items were greater than 0.896, which is above the acceptable limit of 0.5 (Field, 2013). Bartlett’s sphericity test showed statistical significance (χ^2^ [136] = 8027.578; *p* < 0.001).

The Guttmann–Kaiser’s criterion revealed three components with eigenvalues over one that explained 61.42% of the total variance. Cattel’s scree test was in line with Guttmann–Kaiser’s criterion and showed gradations that would justify using three components. Horn’s parallel analysis also suggested a three principal component solution with eigenvalues higher than the threshold value taken out from the equally large matrix of random numbers – 17 variables ^∗^ 860 respondents. In line with all these analyses, we retained three factors. **Table 1** shows the factor loadings after rotation. The first component is a combination of dedication and absorption that point to intrinsic motivation and involvement in one’s job. The original UWES dedication items all load on the first component. The second component is the combination of vigor and absorption, indicating fortitude and general activity associated with the job. We can see that the original vigor and absorption dimensions split into two components each. The third component is loaded only by vigor items that point to stamina while the rest of the vigor items that load on the second component point to fortitude.

All 9 items of the UWES-9 were subjected to PCFA. First, data were analyzed in order to estimate adequacy for the PCFA. The value of KMO measure was 0.915 and all KMO values for the individual items were greater than 0.878. Bartlett’s sphericity test showed statistical significance (χ^2^[36] = 4758.681; *p* < 0.001). The Guttmann–Kaiser’s criterion revealed one component with eigenvalues over one that explained 60% of the total variance (Eigenvalue = 5.31). Both Cattel’s scree test and Horn’s parallel analysis (9 ^∗^ 860) were in line with Guttmann-Kaiser’s criterion, justifying the one-component solution. **Table 2** shows the summary of the PCFA for the UWES-9 scale – the correlations with the first principal component, and variance explained by the component. Contrary to the original three-dimensional model of the short, 9-item UWES scale, the analysis of the ratings of the Serbian sample produced a one-factor solution.

The UWES-17 and UWES-9 factorial structures were tested with the CFA. Questionnaires with missing data were excluded from the analysis. Based on Bakker and colleagues’ original model (Schaufeli and Bakker, 2003), we tested both the 17-item and 9-item versions for the one and three-factor models. Additionally, based on exploratory factor analysis of the Serbian data, we tested the three-factor model for the UWES-17 scale.

The goodness of fit indices for the tests of factorial validity of the UWES-17 and UWES-9 models are presented in **Table 3**. Since the values of CMIN/df were in the range up to 5 for almost all tested models, they were acceptable according to Wheaton et al. (1977). Only the UWES-9 one-factor model did not produce the acceptable CMIN/df value. Based on the RMSEA cut-off value of 0.08, all tested models indicate a mediocre fit (MacCallum et al., 1996). As an absolute measure of fit, the standardized root mean square residual (SRMR) of 0.08 indicates a good fit (Hu and Bentler, 1999). This means that the models based on the 17-item scale produce a good fit, whereas testing the 9-item version did not produce an acceptable fit, either as a one-factor or as a three-factor solution. The CFI, GFI and AGFI did not reach desirable values close to 1 (Blunch, 2013). However, GFI values were closest to a good fit.

**Table 3:** Goodness of fit indices for tests of factorial validity of the UWES-17 and UWES-9 models.

Model	No. of	χ*2*	*df*	*p*	*CMIN/df*	*RMSEA*	*SRMR*	*CFI*	*GFI*	*AGFI*
	items					[90% CI]				
One factor	17	465.565	119	0.000	3.912	0.059 [0.053 -0.064]	0.0785	0.695	0.920	0.897
Three factor – Serbian structure	17	415.349	116	0.000	3.581	0.055 [0.049 -00.061]	0.0695	0.736	0.929	0.906
Three factor – original structure	17	420.498	116	0.000	3.625	0.056 [0.050 -0.061]	0.0766	0.732	0.928	0.905
One factor	9	144.957	27	0.000	5.369	0.072 [0.060 -0.083]	0.0831	0.832	0.951	0.918
Three factor – original version	9	116.546	24	0.000	4.856	0.067 [0.055 -00.080]	0.1002	0.868	0.960	0.926


**CMIN/df*, Chi-square/degrees of freedom; *RMSEA*, Root Mean Square Error of Approximation; *SRMR*, Standardized Root Mean Square Residual; *CFI*, Comparative Fit Index; *GFI*, Goodness-of-Fit Index; *AGFI*, Adjusted Goodness-of-Fit Index.*

Descriptive analyses including means, standard deviations, values of skewness and kurtosis and Cronbach’s alpha coefficients of the UWES-17 and UWES-9 versions with the corresponding dimensions of vigor, dedication and absorption and the Serbian dimensions based on the UWES-17 factorial structure are presented in **Table 4**. Cronbach’s alpha scores indicated high internal consistency of all checked scales and subscales, except for the subscale “absorption” on the UWES-9 scale. The skewness and kurtosis values indicate that the distributions of all tested scales could have been regarded as normal, except the subscale “absorption” on the UWES-9.

**Table 4:** Means, standard deviations, skewness and kurtosis values, and Cronbach’s alphas of the UWES Serbian adaptation scales and dimensions.

	No. of items	*M*	*SD*	*Skewness (SE)*	*Kurtosis (SE)*	*Cronbach’s alpha*
UWES-17	17	4.01	0.97	-0.459 (0.08)	0.056 (0.17)	0.924
UWES-17, vigor	6	3.91	0.96	-0.361 (0.08)	0.393 (0.17)	0.799
UWES-17, dedication	5	4.19	1.25	-0.644 (0.08)	-0.078 (0.17)	0.872
UWES-17, absorption	6	3.94	1.03	-0.377 (0.08)	0.469 (0.17)	0.787
UWES-17, SF1	8	4.25	1.11	-0.634 (0.08)	-0.008 (0.17)	0.901
UWES-17, SF2	6	3.60	1.13	-0.291 (0.08)	0.120 (0.17)	0.838
UWES-17, SF3	3	4.16	1.00	-0.419 (0.08)	0.250 (0.17)	0.650
UWES-9	9	3.97	1.11	-0.419 (0.08)	-0.169 (0.17)	0.904
UWES-9, vigor	3	3.66	1.24	-0.375 (0.08)	-0.258 (0.17)	0.846
UWES-9, dedication	3	4.04	1.36	-0.604 (0.08)	-0.081 (0.17)	0.874
UWES-9, absorption	3	4.23	1.13	0.262 (0.08)	6.167 (0.17)	0.623


*SF1, Factor 1 from this research (**Table 2**); SF2, Factor 2 from this research (**Table 2**); SF3, Factor 3 from this research (**Table 1**); VI, Vigor; DE, Dedication; AB, Absorption.*

The inter-correlations of the UWES long and short scale versions and their component scales, including the Serbian PCFA solution, are presented in **Table 5**. As expected, the majority of observed correlations are very strong (above 0.70). The highest correlation is among long and short forms’ totals. The totals of both forms strongly correlate with their original component subscales. The correlations of Serbian component scales with the scale total are very strong for the first two components and strong for the third component. The long and the short UWES corresponding sub-scales share more than 79% of variance.

**Table 5:** Inter-relationships among work engagement three-dimensional and one-dimensional models based on the UWES-17 and UWES-9.

	UWES-17	UWES-17	UWES-17	UWES-9	UWES-17	UWES-17	UWES-17	UWES-9	UWES-9	UWES-9
	SF2	SF3			VI	DE	AB	VI	DE	AB
UWES-17, SF1	0.755^∗∗^	0.509^∗∗^	0.940^∗∗^	0.920^∗∗^	0.747^∗∗^	0.961^∗∗^	0.841^∗∗^	0.748^∗∗^	0.937^∗∗^	0.772^∗∗^
UWES-17, SF2		0.519^∗∗^	0.910^∗∗^	0.921^∗∗^	0.864^∗∗^	0.738^∗∗^	0.878^∗∗^	0.923^∗∗^	0.771^∗∗^	0.781^∗∗^
UWES-17, SF3			0.670^∗∗^	0.537^∗∗^	0.823^∗∗^	0.446^∗∗^	0.564^∗∗^	0.470^∗∗^	0.443^∗∗^	0.533^∗∗^
UWES-17				0.970^∗∗^	0.906^∗∗^	0.900^∗∗^	0.915^∗∗^	0.866^∗∗^	0.900^∗∗^	0.832^∗∗^
UWES-9					0.860^∗∗^	0.897^∗∗^	0.880^∗∗^	0.902^∗∗^	0.927^∗∗^	0.850^∗∗^
UWES-17, VI						0.717^∗∗^	0.761^∗∗^	0.888^∗∗^	0.733^∗∗^	0.684^∗∗^
UWES-17, DE							0.725^∗∗^	0.751^∗∗^	0.967^∗∗^	0.664^∗∗^
UWES-17, AB								0.724^∗∗^	0.742^∗∗^	0.912^∗∗^
UWES-9, VI									0.781^∗∗^	0.631^∗∗^
UWES-9, DE										0.679^∗∗^


*^∗∗^Correlation is significant at the 0.01 level.*

### Work Engagement and Well-being

In order to explore the validity of the tested models of work engagement we analyzed the relationships of work engagement models with the selected indicators of well-being, i.e., burnout, organizational support, job satisfaction, and job insecurity. Pearson product-moment correlations between work engagement and well-being indicators (burnout, organizational support, job satisfaction and job insecurity) are presented in **Table 6**. It should be noted that correlation analyses were performed on different subsamples, thus the sizes of the samples differ. The general tendency is that the long and short forms of the UWES Serbian adaptation produce similar correlations with well-being indicators. The total scores both for the long and short versions produced the strongest correlations. Among indicators, the total score for job burnout was the strongest correlate, whereas job insecurity was the lowest correlate of work engagement. At the level of engagement dimensions, the original UWES-17 solution produced higher correlations than the solution suggested based on exploratory factor analysis of the Serbian sample data. Although PCFA of the UWES-9 produced a one-factor solution (**Table 2**), inter-correlations of the short version original factors with well-being measures are similar to those based on the 17-item version factors that suggest the UWES-9 dimensions could still develop meaningful results.

**Table 6:** Pearson product-moment correlations between work engagement (three-dimensional and one-dimensional and models based on the UWES-17 and UWES-9) and well-being indicators (burnout, organizational support, job satisfaction and job insecurity).

	Burnout total	Burnout exhaustion	Burnout disengagement	Organizational support	Job satisfaction	Job insecurity
	
*N*	84	84	84	290	219	577
UWES-17	-0.758^∗∗^	-0.583^∗∗^	-0.723^∗∗^	0.642^∗∗^	0.686^∗∗^	0.241^∗∗^
UWES-17, VI	-0.691^∗∗^	-0.556^∗∗^	-0.597^∗∗^	0.607^∗∗^	0.631^∗∗^	0.239^∗∗^
UWES-17, DE	-0.728^∗∗^	-0.564^∗∗^	-0.721^∗∗^	0.631^∗∗^	0.656^∗∗^	0.237^∗∗^
UWES-17, AB	-0.649^∗∗^	-0.470^∗∗^	-0.653^∗∗^	0.500^∗∗^	0.605^∗∗^	0.161^∗∗^
UWES-17, SF1	-0.710^∗∗^	-0.520^∗∗^	-0.736^∗∗^	0.609^∗∗^	0.635^∗∗^	0.219^∗∗^
UWES-17, SF2	-0.704^∗∗^	-0.590^∗∗^	-0.626^∗∗^	0.600^∗∗^	0.662^∗∗^	0.215^∗∗^
UWES-17, SF3	-0.466^∗∗^	-0.323^∗∗^	-0.380^∗∗^	0.383^∗∗^	0.448^∗∗^	0.154^∗∗^
UWES-9	-0.753^∗∗^	-0.588^∗∗^	-0.728^∗∗^	0.657^∗∗^	0.683^∗∗^	0.243^∗∗^
UWES-9, VI	-0.709^∗∗^	-0.613^∗∗^	-0.632^∗∗^	0.621^∗∗^	0.659^∗∗^	0.243^∗∗^
UWES-9, DE	-0.721^∗∗^	-0.565^∗∗^	-0.712^∗∗^	0.637^∗∗^	0.667^∗∗^	0.251^∗∗^
UWES-9, AB	-0.634^∗∗^	-0.423^∗∗^	-0.657^∗∗^	0.503^∗∗^	0.507^∗∗^	0.140^∗∗^


*^∗∗^ Correlation is significant at the 0.01 level.*

To examine whether work engagement explained incremental variance above job burnout, we performed hierarchical multiple regression analysis (with both the UWES-17 total scores and the UWES-9 total scores) with job satisfaction as an outcome variable. Hierarchical regression analysis for the effect of burnout and work engagement on job satisfaction is presented in **Table 7**, for the cases when engagement was assessed by the UWES-17, and in **Table 8** for the cases when engagement was assessed by the UWES-9. Demographic variables were not included as their relations with work engagement were not considered meaningful (**Table 9** and next section). The first step included the OLBI subscales Burnout exhaustion and Burnout disengagement, and the second step added the UWES-17 total score (**Table 7**) or the UWES-9 total score (**Table 8**). The UWES total scores were used because of the high inter-correlations among the UWES subscales (**Table 5**).

**Table 7:** Hierarchical regression analyses for the effect of burnout (exhaustion and disengagement) and work engagement assessed by the UWES-17 on job satisfaction.

	*R*^2^	Adj.*R*^2^	*R*^2^ change	*B*	Beta	*F*(df1, df2)
**Model 1**						
Burnout exhaustion	0.493	0.480		-0.037	-0.315^∗∗^	38.90 (2, 80)
Burnout disengagement				-0.052	-0.445^∗∗∗^	
**Model 2**						
Burnout exhaustion	0.563	0.547	0.070^∗∗^	-0.030	-0.255^∗^	33.98 (3, 79)
Burnout disengagement				-0.024	-0.206	
UWES-17 Total				0.024	0.389^∗∗^	


*^∗^*p* < 0.05, ^∗∗^*p* < 0.01, ^∗∗∗^*p* < 0.001.*

**Table 8:** Hierarchical regression analyses for the effect of burnout (exhaustion and disengagement) and work engagement assessed by the UWES-9 on job satisfaction.

	*R*^2^	Adj.*R*^2^	*R*^2^ change	*B*	Beta	*F*(df1, df2)
**Model 1**						
Burnout exhaustion	0.493	0.480		-0.037	-0.315^∗∗^	38.90 (2, 80)
Burnout disengagement				-0.052	-0.445^∗∗∗^	
**Model 2**						
Burnout exhaustion	0.559	0.542	0.066^∗∗^	-0.030	-0.256^∗^	33.41 (3, 79)
Burnout disengagement				-0.025	-0.210	
UWES-9 Total				0.038	0.380^∗∗^	


*^∗^*p* < 0.05, ^∗∗^*p* < 0.01, ^∗∗∗^*p* < 0.001.*

**Table 9:** Means and standard deviations of the UWES-17 and UWES-9 total scores across demographic subgroups.

		Gender		Education			Hierarchical position		Ownership of the organization	
					
		Men	Women	High school	Trade school, college	University, undergraduate and more	Subordinate	Superior	State	Private
UWES-17	*M* (*SD*)	65.63 (17.89)	69.55 (15.59)	65.57 (17.81)	69.57 (14.80)	70.35 (14.90)	67.19 (16.74)	71.29 (14.93)	68.69 (15.71)	66.51 (17.50)
UWES-9	*M* (*SD*)	33.86 (10.76)	36.96 (9.40)	34.25 (10.52)	36.73 (9.23)	37.42 (9.67)	35.19 (10.14)	37.93 (8.90)	36.42 (9.29)	34.48 (10.76)


In both cases, when the UWES was included it gave better prediction of job satisfaction (**Tables 7**, **8**). The UWES-17 total explained 7% of the variance in job satisfaction, while the UWES-9 total explained 6.6% of the job satisfaction variance. It is also notable that adding the UWES (in both analyses) lowers the coefficients of burnout disengagement and makes them insignificant. Taken together, the results demonstrate the predictive value of the UWES.

### Work Engagement in Different Groups of Employees

Means and standard deviations of the UWES-17 and UWES-9 total scores across demographic subgroups are presented in **Table 9**. Comparing women and men on the UWES total scores revealed that women were more engaged than men, both on the long form [*F*(1,846) = 11.145; *p* = 0.001, η^2^ = 0.013] and short form [*F*(1,848) = 19.301; *p* < 0.001, η^2^ = 0.022]. The UWES scores had significant but low correlations with employees’ age, both for the long (*r* = 0.157, *p* < 0.001) and short form (*r* = 0.164, *p* < 0.001). Higher educated employees were more engaged [long form: *F*(2,751) = 7.340; *p* = 0.001, η^2^ = 0.020; short form: *F*(2,753) = 8.910; *p* < 0.001, η^2^ = 0.023]. Employees on supervisory positions were more engaged than others [long form: *F*(1,829) = 6.186; *p* = 0.013, η^2^ = 0.010; short form: *F*(1,831) = 7.592; *p* = 0.006, η^2^ = 0.010]. Employees working at state-owned organizations were more engaged than those working at privately owned companies based on the UEWS short form [long form: *F*(1,822) = 3.551; *p* = 0.060, η^2^ = 0.004); short form: *F*(1,824) = 7.704; *p* = 0.006, η^2^ = 0.010].

## Discussion

The overall aim of the present study was to provide evidence of the psychometric properties of the Serbian versions of the UWES, the long and short one, by exploring their factor structure, internal consistency and criterion validity.

A detailed analysis confirmed that both the UWES-17 and UWES-9 were applicable in the Serbian cultural context. Based on the UWES-17 findings, our data have confirmed both the three-factor and one-factor solutions, giving a slight advantage to the three-factor solution. As for the UWES-9, based on the PCFA and CFA findings, we obtained one-factor as a preferred solution. Based on a systematic review of research papers dealing with the UWES factorial validity within the CFA approach, the one-factor solution of the UWES-9 was also suggested by Kulikowski (2017). Taking into the account all other analyses presented in this paper, we would opt for applying both one and three-factor solutions for the UWES-9 Serbian version.

The PCFA of the 17-item version of the UWES-S showed a three-factor structure with the content somewhat different from the original model. The first component was majorly loaded by dedication items (including absorption items), the second component was loaded by vigor and absorption items, while the third component was clearly loaded only by vigor items. The most “problematic” were the absorption items that actually spread across two dimensions. The first component pointed to involvement in one’s job, the second could indicate general activity, while the third component indicated stamina. The item that was particularly problematic was the DE2 item (“I am enthusiastic about my job”) that loaded almost equally on both the first and the second component. The PCFA of the 9-item version showed the existence of only one component, with loadings above 0.590.

Overall, CFA revealed better fit indices for the UWES-17 than the UWES-9. The long version of the UWES showed the best fit when three factors were taken into account. The fit indices were somewhat better for the structure that was obtained by PCFA, but the indices for the original three factor model were also acceptable. The UWES-9 showed a somewhat better fit when the one-factor solution was tested. Nevertheless, taking into consideration the fit indices of all tested models, we can conclude that we only gained an acceptable, but not a preferred fit. Even though these indices are acceptable when comparing with other international samples (e.g., Schaufeli and Bakker, 2003; Schaufeli et al., 2006), this finding could be also interpreted as an “invitation” for making some culturally more sensitive forms of the UWES. Bearing in mind qualitative data from the focus group discussion about understanding the UWES items, the presented data suggest it is reasonable to question whether the employees from Serbia conceptualize work engagement similarly to Dutch employees. In a broader perspective, obtained results point to the need for more extensive and precise multicultural comparisons, which is in line with Balducci et al.’s (2010) conclusions about the need for more stringent cross-cultural research.

Contrary to the ambiguities brought by PCFA and CFA, the reliabilities, inter-correlations and the pattern of correlations of the UWES (short and long) with the corresponding well-being variables clearly met all theoretical expectations. The reliabilities of the UWES-17 and UWES-9 total scores were higher than 0.90. All the originally postulated subscales on both forms, except for the UWES-9 absorption, showed reliability above 0.78. The inter-correlation between subscales of both forms showed that the corresponding sub-scales shared more than 79% of variance. In addition, the correlation of both UWES forms with burnout, job satisfaction, organizational support and job insecurity showed an almost equal pattern of correlations when analyzing total scores and the scores on particular subscales. Finally, when taking into account the incremental validity of work engagement above job burnout, assessed by both the UWES-17 and the UWES-9, in both cases, when UWES was included, it gave better prediction of job satisfaction with almost equal values of all parameters. These positive and theoretically expected findings indicate that both the UWES-17 and the UWES-9 can be used with equal success in research of work engagement in Serbia.

Observed relationships between work engagement (assessed both by UWES-17 and UWES-9) and demographic variables add to the muddled picture of the previously published correlations that are clearly inconsistent (e.g., Schaufeli et al., 2006; Fong and Ng, 2012; Littman-Ovadia and Balducci, 2013; Lovakov et al., 2017). Generally, when comparing the employees’ levels of engagement across gender, education, hierarchical position, as well as the ownership of organization, it should be noted that observed statistically significant differences have fallen under the shadow of the low effect size. As underlined by Schaufeli et al. (2006), these differences lack practical significance.

The obtained results are more interesting and valuable because they come from the Serbian socio-economic context, in which, for the past quarter of the century, employees have been confronted with privatizations, restructuring, downsizing, closing down and the collapse of a great number of companies (Petrovic and Cizmic, 2010). For many it meant either losing their job or early retirement. Losing a job subsequently led to long-term unemployment and exponentially declining chances of finding a job. As for the employees who stayed at work as the ‘survivors’ of organization and society-wide negative processes, it is questionable whether staying at work sustained their work engagement or eroded it. As noted in our previous research, the mechanisms which helped the workers to cope with the crisis could be a serious threat for readapting to working under the ‘normal’ conditions (Čizmić et al., 1995). The third work engagement component obtained by PCFA, loaded by three vigor items, named ‘stamina,’ could be related to the specificities of the Serbian social and economic context. Further, different factor solutions yielded based on different UWES language versions could be an impulse for exploring cultural specificities of work engagement and searching for culturally invariant factors of the UWES.

Some potential limitations may exist in the presented research. Although we had a large sample from different companies, economy sectors and occupational groups that were chosen to represent Serbian workforce well, the sample was not composed as statistically representative. It can pose the limitation for comparing the presented findings with the findings of other validation studies, more so because the majority of these studies were also not carried out on statistically representative samples. The cross-sectional nature of the study can also be regarded as a limitation for generalizing and fully utilizing the findings that at present do not give grounds for inferring about the directions of the presented relationships. As suggested by other researchers, future longitudinal studies should uncover causal relationships of work engagement with other well-being correlates (e.g., Schaufeli et al., 2006; Shimazu et al., 2008; Fong and Ng, 2012). Moreover, as noted by Littman-Ovadia and Balducci (2013), the restriction of range in this kind of research design could be expected for work engagement and especially in the Serbian work context. The restriction of range should also be taken into account in those countries with high rates of emigrating workforce. Keeping in mind that work engagement is one of the building blocks of the JD–R model, to develop a full understanding and validation of the model, engagement should be more fully explored in the relationship with other features of the model. Finally, as noted by some researchers (e.g., Fong and Ng, 2012), self-report nature of applied measures is prone to common method variance. Thus, future studies could include some more objective measures of work engagement.

## Conclusion

The current study highlights the validity of the UWES in the specific social and economic context of Serbia. The presented results have shown that both the UWES-17 and the UWES-9 Serbian versions have satisfactory psychometric properties with high reliability, factorial structure in line with theoretical model and good predictive validity, thus confirming that these UWES versions are applicable in the Serbian context, both for use in research and for practical purposes. Nevertheless, there is a need for exploring how employees from Serbia as well as other countries perceive work engagement. As suggested by PCA, it is reasonable to question whether the employees from Serbia conceptualize work engagement in the same way as the workers from other cultures and economies. Future cross-cultural research should further investigate the cultural invariance of the UWES factorial structure under more controlled conditions. The presented research could be regarded as a contribution to a positive approach to evolving international economic cooperation. Shedding light on work engagement contributes to a positive psychology approach that is essential for successful work transformation and organizational transitions in globalizing economy.

## Author Contributions

All three authors, IP, MV, and SČ contributed equally to the research design and writing of this paper.

## Conflict of Interest Statement

The authors declare that the research was conducted in the absence of any commercial or financial relationships that could be construed as a potential conflict of interest.

## Footnotes

1 We would like to express gratitude to Jelena Branković and Tamara Jevtić for organizing and moderating focus group discussion.
